# A back-to-back tumor composed of papillary renal cell carcinoma and oncocytoma treated by laparoscopic partial nephrectomy: a case report

**DOI:** 10.1186/s13256-018-1662-7

**Published:** 2018-05-21

**Authors:** Su Hwan Kang, Won Tae Seo, Pil Moon Kang, Hyun Yul Rhew, Yo Han Jeon, Bong Kwon Cheon, Taek Sang Kim

**Affiliations:** Departments of Urology and pathology, Kosin University Gospel Hospital, Kosin University College of Medicine, 262 Gamcheon-ro, Seo-gu, Busan, 49267 South Korea

**Keywords:** Collison tumor, Hybrid tumor, Laparoscopic partial nephrectomy, Papillary renal cell carcinoma, Renal oncocytoma

## Abstract

**Background:**

Renal oncocytoma is the most common benign renal tumor, and papillary renal cell carcinoma is the second most common histologic subtype of renal cell carcinoma. Renal tumors containing different components such as papillary renal cell carcinoma and oncocytoma are extremely rare.

**Case presentation:**

A renal mass was incidentally detected in a 52-year-old Korean woman, and a computed tomographic scan showed a 32-mm multicystic mass with some calcifications in the lower pole of the right kidney. She underwent laparoscopic partial nephrectomy without any perioperative complications. We found a papillary renal cell carcinoma and an oncocytoma in a tumor mass.

**Conclusions:**

The possibility of a mixed malignant tumor should be considered while treating benign tumors such as oncocytoma.

## Background

Renal oncocytoma is the most common benign renal tumor. It appears as an enhancing renal mass on computed tomographic images and is indistinguishable from renal cell carcinoma (RCC). It accounts for 3% to 7% of all kidney tumors [1]. It is derived from intercalated cells of distal renal tubules. Papillary renal cell carcinoma (PRCC) is the second common histologic subtype of RCC, and it accounts for 10% to 15% of all RCCs [2]. PRCC can be divided into subtypes 1 and 2; subtype 2 is a more aggressive subtype [3]. PRCC arises from proximal renal tubules. Occasionally, it has been reported that hybrid tumors of the kidney contain chromophobe RCC and oncocytoma, because they are a closely related spectrum of neoplasms, although one is benign and the other is malignant. However, other hybrid tumors of the kidney are very rare, and we report a case of a patient with a back-to-back tumor that contained oncocytoma and PRCC components.

## Case presentation

In December 2015, an asymptomatic 52-year-old Korean woman with hypertension was referred to our hospital because of a right-sided renal mass detected on transabdominal ultrasound. She was a housewife and did not have any medical history. Her physical examination was unremarkable, and routine laboratory studies did not document any abnormalities (creatinine 0.47 mg/dl, normal complete blood count, normal liver function test, normal serum electrolyte concentrations, microscopic hematuria 2+).

**Fig. 1:**
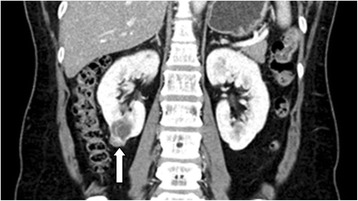
Location of the tumor visualized by computed tomography. The *arrow* shows a well- marginated, multiseptated mass in the lower pole of the right kidney

Computed tomography (CT) of the abdomen demonstrated a 32-mm multicystic mass with some calcifications in the lower pole of the right kidney (Fig. 1). At the lower end of the mass, a small additional mass was detected, but it was thought to be part of the multicystic mass. The patient underwent laparoscopic partial nephrectomy. The bullous tumor was found in the operative field at the lower pole of the right kidney. The tumor was marked by a monopolar hook electrode, and it was excised using 10-mm Metzenbaum scissors with minimal safety margins. Clamping of the renal artery was performed, followed by gentle resection with a harmonic blade. After resection of the tumor, it was confirmed that the kidney calyx was opened. The opened calyx and remnant renal parenchyma were closed with a V-Loc™ (Covidien, Dublin, Ireland) and 3-0 Vicryl suture (Ethicon, Somerville, NJ, USA). The clamping time was 27 minutes. The patient was discharged 7 days after surgery without any postoperative complications. She did not receive chemotherapy or any other treatment after surgery, and we have been following her for 2 years.

**Fig. 2:**
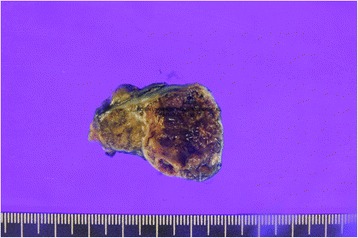
Grossly, the mass was divided into two small masses (right and left). The oncocytoma (*left*) was a tan, homogeneous, white yellowish mass with a central scar. The papillary renal cell carcinoma (*right*) was well circumscribed with a pseudocapsule, a dark brown color, and a spherical boundary

Grossly, the ovoid mass was well circumscribed and measured 3.5 × 2.7 × 2.5 cm, and on sections, the mass was composed of two back-to-back masses (Fig. 2). The mass was 0.7 mm and 0.5 mm away from the renal capsular margin and the renal parenchymal margin, respectively. The larger mass was cystic, and it was filled with tan necrotic material. The smaller mass was bright brown and solid with numerous tiny cystic spaces. The larger mass measured 2.6 × 2.5 cm in cross-sectional diameter, and the smaller mass was 1.5 × 1.5 cm in cross-sectional diameter.

**Fig. 3:**
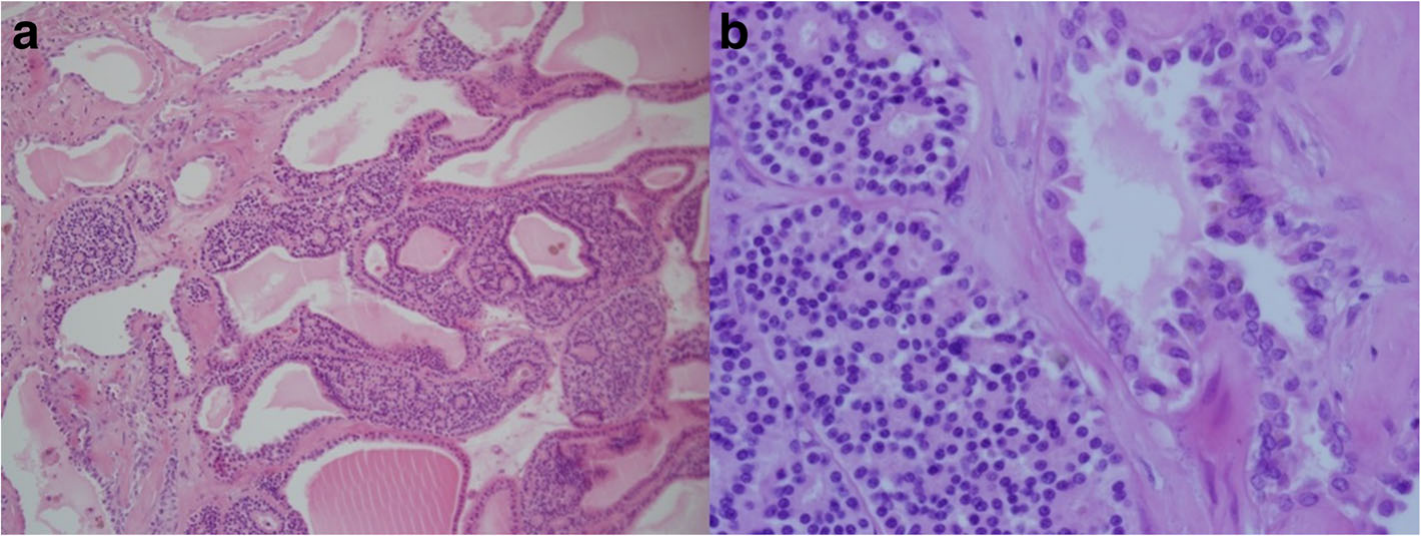
**a** The renal oncocytoma component is in the *central area* was composed of large eosinophilic cells with a cyst of variable size visualized by hematoxylin-eosin (H&E) staining (left, original magnification × 40). **b** *Left half* was the oncocytoma component and *right half* was the papillary renal cell carcinoma component visualized by H&E staining (right, original magnification × 400)

Microscopically, the smaller mass showed solid nest architecture of low cuboidal eosinophilic cells admixed with cystic spaces lined by low cuboidal eosinophilic cells (Fig. 3). These epithelial cells had abundant, densely granular cytoplasm and uniform, round, bland nuclei with no mitoses. There was a focal area of tubular or cystic structures lined by atypical epithelial cells with a hobnail appearance. Focally, these atypical tubules or cysts were admixed with the oncocytoma component. A few cystic spaces were lined by oncocytes that abruptly continued with these atypical epithelial lining cells. These atypical epithelial cells had round, ovoid, or occasionally cleaved nuclei and small to moderate amounts of eosinophilic or occasionally clear cytoplasm. The size of the nuclei of these atypical cells was 1.5–2 times that of the nuclei of the onocytoma, with an increased nuclear/cytoplasmic ratio and visible or prominent nucleoli. There were occasionally papillary protrusions in the atypical tubules or cystic spaces. Immunohistochemical staining showed that the cells of the oncocytoma were immunopositive for CD117 but negative for cytokeratin 7, vimentin, CD10, and α-methylacyl-coenzyme A racemase (AMACR). The atypical tubular and cystic epithelial cells were immunopositive for cytokeratin 7 and vimentin and focally positive for CD10, but they were negative for CD117 and AMACR. The histological and immunohistochemical results were consistent with a hybrid neoplasm composed of PRCC and oncocytoma.

The preoperative creatinine level was 0.47 mg/dl, and the estimated glomerular filtration rate (eGFR) calculated by the Modification of Diet in Renal Disease equation was ≥ 120 ml/minute/1.73m^2^. At the 12-month follow-up after surgery, the patient’s creatinine level was 0.52 mg/dl, and her eGFR was ≥ 120 ml/minute/1.73m^2^. There was no evidence of recurrence on chest or abdominal CT.

## Discussion

We report the characteristics of a hybrid tumor of the kidney. This tumor is occasionally called a “collision tumor.” The term *collision tumor* refers to coexistent but independent tumors that are histologically distinct [4]. Collision tumors in the kidney have been described, and they mostly have included oncocytoma and chromophobe RCC. Hereditary disease such as Birt-Hogg-Dubé syndrome shows coexistence of oncocytoma and other RCCs of an origin similar to that of chromophobe RCC [5]. PRCC and oncocytoma have different cellular origins, and therefore such a hybrid tumor is very rare.

To the best of our knowledge, about six other case reports have been reported to date, which makes our patient’s case the seventh. The previously reported cases are summarized in Table 1. Unlike the other reported patients, our patient was relatively young at 52 years old, and she had a high Fuhrman grade of 3. In all of the previous cases, oncocytoma was larger than PRCC. Our patient’s case was an exception. Microscopically, the PRCC portion expanded to the oncocytoma portion, and therefore the oncocytoma portion showed mixed characteristics of PRCC and oncocytoma.

**Table 1:** Previously reported cases of hybrid tumor composed of papillary renal cell carcinoma and oncocytoma

Study	Age (years)/sex	PRCC size (cm)	Fuhrman grade	PRCC subtype	Treatment	Follow-up outcome
Rowsell *et al*. [6]	75/M	0.7	2	1	P	NA
Floyd *et al*. [7]	73/F	1.5	NA	2	P	NA
Sejben *et al*. [8]	68/M	1.0	2	2	R	8
Vasuri *et al.* [9]	69/M	NA	NA	NA	?	NA
Ozer *et al.* [10]	74/M	1.7	NA	2	R	18
Goyal *et al.* [11]	78/M	1.0	2	?	P (hand-assisted)	28
Our patient	52/F	2.6	3	1	LP	13

*Abbreviations: P* Partial nephrectomy, *R* Radical nephrectomy, *LP* Laparoscopic partial nephrectomy, *NA* Not available, *PRCC* Papillary renal cell carcinoma

## Conclusions

Oncocytoma is a common benign tumor, and occasionally a small renal mass such as a benign tumor is treated with active surveillance or ablation. We treated this ambiguous tumor by laparoscopic partial nephrectomy. The possibility of a mixed malignant tumor should be considered while treating benign tumors such as oncocytoma.
